# Cost-consequence analysis of salvianolate injection for the treatment of coronary heart disease

**DOI:** 10.1186/s13020-018-0185-x

**Published:** 2018-06-14

**Authors:** Pengxin Dong, Hao Hu, Xiaodong Guan, Carolina Oi Lam Ung, Luwen Shi, Sheng Han, Shuwen Yu

**Affiliations:** 1 School of Pharmaceutical Sciences, Shandong University, Jinan, Shandong China; 2 International Research Center of Medical Administration, Peking University, Beijing, China; 3 State Key Laboratory of Quality Research in Chinese Medicine, Institute of Chinese Medical Sciences, University of Macau, Taipa, Macao; 4 School of Pharmaceutical Science, Peking University Health Science Center, Beijing, China; 5 Shandong University Affiliated Jinan Central Hospital, Jinan, Shandong China

**Keywords:** Cost-consequence analysis, Salvianolate injection, Coronary heart disease, Chronic ischemic heart disease, Traditional Chinese medicine, Pharmacoeconomics

## Abstract

**Background:**

Complicated with the impact of aging population and urbanization, coronary heart disease (CHD) incurs more and more disease burdens in China. Salvianolate injection is a Chinese patent drug widely used for treating CHD in China. A series of studies have verified the efficacy of salvianolate injection , but the high drug cost has raised concerns. It is, therefore, important to conduct cost-consequence analysis to demonstrate whether salvianolate injection is associated with outcome improvement and cost containment. The aim of this study was to retrospectively evaluate the cost-consequence of salvianolate injection for the treatment of coronary heart disease by combining salvianolate injection with conventional treatment from a societal perspective.

**Methods:**

We retrospectively studied hospitalized patients with CHD from August 2011 to December 2015 by using electronic medical record database. Patients who received salvianolate injection combined with conventional treatment were selected as exposed group, while those who received conventional treatment alone were selected as unexposed group. Propensity score matching (PSM) analysis was used to balance the characteristics of patients. After PSM, we evaluated hospital stay, total nitrates dosage, total medical costs, and subcategories costs. Patients with chronic ischemic heart disease were analyzed as a highly selected subcohort.

**Results:**

For the overall group, hospital stay was significantly decreased by 2.9 days (*P* < 0.05) and total nitrates dosage was significantly decreased by 172.4 mg (*P* < 0.05) in exposed group; cost savings of pharmacy cost, examination cost, laboratory cost, operation cost and treatment was observed as significant (at *P* < 0.05); and the additional expenditure of Chinese patent drug (1174.9 CNY) was less than the saving of total medical costs (2636.4 CNY). For chronic ischemic heart disease subcohort, compared with unexposed group, significant decreases were also found in hospital stay and total nitrates dosage (*P* < 0.05); cost savings were significant (*P* < 0.05) for exposed group in terms of total medical costs (4339.5 CNY) and subcategories costs (including pharmacy cost, examination cost, operation cost and treatment cost); and the additional expenditure of Chinese patent drug (1189.3 CNY) was less than the saving of total medical costs.

**Conclusion:**

Compared with conventional treatment for the treatment of CHD, combination of salvianolate injection and conventional treatment was associated with a reduction in hospital stay and total nitrates dosage. The acquisition cost of Chinese patent drug (including salvianolate injection) was offset by a higher reduction in total medical costs, especially for chronic ischemic heart disease.

**Electronic supplementary material:**

The online version of this article (10.1186/s13020-018-0185-x) contains supplementary material, which is available to authorized users.

## Background

Coronary heart disease (CHD) incurs great costs and heavy economic burdens worldwide [1, 2]. According to the findings from the Global Burden of Disease Study 2013, 92.94 million people were suffering from this disease [3]. As for China, complicated with the impact of aging population and urbanization, CHD prevalence and mortality increased sharply during the past few decades [4, 5]. In 2014, a total of 500,946 Chinese patients received percutaneous coronary intervention due to CHD, causing high medical expenditure for CHD patients in China [6].

Based on the guidelines introduced by the National Health and Family Planning Commission of the People’s Republic of China, traditional Chinese medicine (TCM) is considered one of the general treatment for CHD [7]. TCM regards CHD as “thoracic obstruction”. The primary TCM syndrome of CHD is blood stasis. Therefore, the major objectives of TCM therapy is to promote blood circulation, remove blood stasis and dredge collaterals [8, 9]. Comparing with western medicine, TCM functions through multiple paths and multiple target spots, so that it is always expected to improve patients’ overall health status [10].

Salvianolate injection is a TCM injection made from the root of a Chinese herb called red-rooted salvia. The indication of salvianolate injection is CHD with stable angina pectoris. The syndrome differentiation in TCM of salvianolate injection is blood stasis syndrome, usually occurring in CHD, arrhythmia and congestive heart failure. Pharmacological studies indicated that salvianolate injection could protect cardiomyocytes via reducing the levels of proinflammatory cytokines and inhibiting reactive oxygen species production [11, 12]. A systematic review made by Zhang [13] in 2016 showed that the combined use of salvianolate injection and western medicine improved both the total effective rate and the electrocardiogram effective rate. Safety of salvianolate injection was confirmed in an overview of systematic reviews made by Liu [14]. In that study, adverse reaction rate of salvianolate injection was considered equivalent to conventional treatment and lower than other TCM injections. Given its favorable efficacy and safety, doctors in China usually supplement conventional treatment with salvianolate injection for CHD patients [15, 16]. According to a ranking list of TCM for cardiovascular and cerebrovascular diseases in March 2016, salvianolate injection had the highest market share in five of the seven Chinese biggest cities [17].

However, the high costs of salvianolate injection has raised concerns. It is, therefore, important to conduct cost-consequence analysis to demonstrate whether salvianolate injection is associated with outcome improvement and cost containment. As shown in the current literature, some studies had tried to conduct economic evaluation of salvianolate injection for CHD. A prospective study to compare cost-consequence of salvia injection, tanshinone II A injection, salvianolate injection, and salvia Kawashima injection for coronary heart disease angina, found salvianolate injection had better cost-consequence [18]. Another study also compared the cost-consequence of these four types of injections and found salvianolate injection showed comparative advantages in term of cost-consequence [19]. However, the above studies only included drug cost and infusion cost without comprehensive assessment of direct medical cost of salvianolate injection for CHD. In addition, they did not consider the impact of salvianolate injection use on possible cost saving on western medicine cost and surgery cost, etc.

The aim of this study was to retrospectively evaluate the cost-consequence of salvianolate injection for the treatment of CHD by combining salvianolate injection with conventional treatment from a societal perspective. Using the medical record database from real world, it is expected to provide references for doctors and policymakers when they need to make clinical or policy decisions about salvianolate injection. Moreover, this study is expected to contribute to the field of pharmacoeconomics of TCM products which is still at the very early development stage in need of further exploratory investigations.

## Methods

### Study population

To realize the research objective, a retrospective cohort study was applied. The minimum standards of reporting checklist of detailed information about experimental design, and statistics, and resources was used in this study (see Additional file 1).

The study cohort was drawn from the electronic medical record database of the Anyang People’s Hospital. The hospital is a 1500-bed, university affiliated tertiary hospital, which is a regional medical center in Henan Province. This study was conducted in 2016 using data from the electronic medical record database since its implementation in August 2011. To collect as many samples as possible, we retrospectively reviewed the inpatients who were admitted to hospital between August 2011 and December 2015. For each sample, the data was retrieved from the day of hospital admission until the day of hospital discharge or hospital death.

In order to extract the study population comprehensively, samples were recruited based on a wide range of diagnosis. Eligible patients included those with ICD-10 code I20 for angina pectoris, or I25 for chronic ischemic heart disease, or description of “angina”, “coronary heart disease”, “coronary artery”, “myocardial infarction”, “heart failure”, “myocardium”, “atrial fibrillation” or “palpitation” in their admitting diagnosis. After preliminary extraction, we performed a manual check to ensure all of study samples were patients with CHD. Patients who received other TCM except salvianolate injection during their hospital stay were excluded from the study.

Among all types of CHD, chronic ischemic heart disease is the most widespread subcategory of CHD. Therefore, we also selected patients with chronic ischemic heart disease in both exposed group and unexposed group as a highly selected subcohort. This subcohort included patients with ICD-10 code I25 or description “coronary heart disease” or “coronary artery”.

### Exposure and outcomes

In the study cohort, patients who received salvianolate injection combined with conventional treatment were selected as exposed group, while those who received conventional treatment alone were selected as unexposed group. According to the medication guide of CHD, conventional treatment included β-blockers, nitrates, calcium channel blockers, lipid regulating agents, anticoagulants and antiplatelet agents, ACE inhibitors and angiotensin receptor blockers. In this study, conventional treatment referred to the treatment included neither salvianolate injection nor other TCM products.

The study outcomes were hospital stay, total nitrates dosage, total medical costs and subcategories costs. At the beginning, we divided total medical costs into 13 subcategories costs: supplies cost, pharmacy cost, examination cost, laboratory cost, operation cost, treatment cost, Chinese patent drug cost, bed cost, nursing cost, blood cost, Chinese herb cost, diet cost, and others cost. Costs composition analysis indicated that among 13 subcategories costs, the accumulative constituent ratio of supplies cost, pharmacy cost, examination cost, laboratory cost, operation cost, treatment cost and Chinese patent drug cost was over 90%. Therefore, we incorporated these seven subcategories costs into final analysis.

Except the outcome measurement above, we also collected the following characteristics of patients: age, sex, diastolic blood pressure, systolic blood pressure, whether or not the patient had joined a medical reimbursement program, nitrates dosage, and administration of nitrates used prior to salvianolate injection.

### Propensity score matching for bias

The major bias in the present study was “selection bias”. In particular, if physicians tended to prescribe salvianolate injection for patients in worse conditions, the cost-consequence would be underestimated. On the contrary, if physicians tended to prescribe salvianolate injection for patients in milder conditions, the cost-consequence would be overestimated. We performed a 1:1 propensity score matching (PSM) analysis to reduce bias [20, 21].

Considering medication background of CHD and data accessibility, we selected age, sex, blood pressure, whether or not the patient had joined a medical reimbursement program as covariates for elementary matching, caliper set at 0.05.

Then, in order to evaluate total nitrates dosage impartially, nitrates dosage and administration of nitrates used prior to salvianolate injection were selected as covariates for further matching, caliper set at 0.01. We selected nitrates dosage and administration of nitrates used prior to salvianolate injection as covariates because nitrates use was an important indicator of the severity of CHD.

In this study, we regarded nitrates use as not only a baseline characteristic but also a clinical outcome. For exposed group, with the first exposure point of salvianolate injection used, the previous line of nitrates exposure was used as a baseline, followed by the use of nitrates as an outcome. For unexposed group, because the first exposure time point of salvianolate injection could not be determined, so we calculated the time for each patient in the exposure group from admission to the first use of salvianolate injection as time interval. Then, for patients in unexposed group, nitrates use during the corresponding time interval was set as a baseline; and nitrates use post time interval was set as an outcome.

**Fig. 1:**
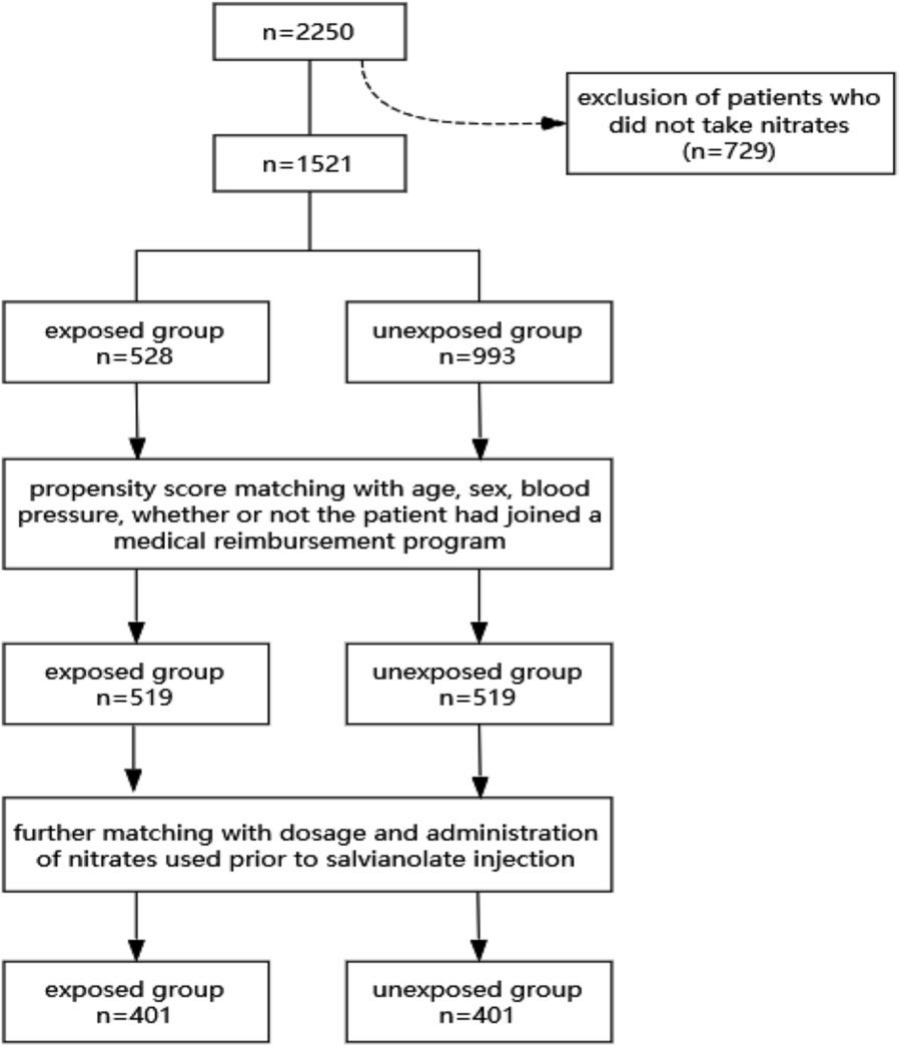
Flow chart of propensity score matching

The whole flow chart of PSM was summarized as Fig. 1.

### Statistical analysis

All analyses were performed using R3.3.3 software. First, descriptive analysis was conducted. Numerical variables were expressed as mean (± standard deviation [SD]). Categorical variables were expressed as percentage.

To test the exposure effects, we performed paired *t* test to assess differences between exposed group and unexposed group. Due to existence of null value, subcategories costs were analyzed with *t* test. A 2-tailed *P* value < 0.05 was considered as significant.

Because this study used PSM to select data, sensitivity analysis was performed for data selection and matching methods. In addition, different statistical test methods were used in this study to verify the sensitivity of the statistical test results to the statistics method.

## Results

### Propensity score matching

First, 2250 patients were collected from the hospital. Second, 729 patients who did not take nitrates were excluded. Third, a total of 802 patients were successfully matched after PSM.

The baseline characteristics of patients before PSM was summarized in Table 1. There were significant (*P* < 0.05) differences in age, diastolic blood pressure, systolic blood pressure, and medical reimbursement between exposed (conventional treatment + salvianolate injection) group and unexposed (conventional treatment + salvianolate injection) group.

**Table 1:** Baseline characteristics before propensity score matching

Characteristic	Conventional treatment + salvianolate injection (n = 635)	Conventional treatment (n = 1615)	*P*
Age (years)	63.3 (± 13.01)	60.4 (± 18.01)	< 0.05
Male sex (%)	50	50	0.414
Diastolic blood pressure (mmHg)	71.1 (± 6.55)	73 (± 7.84)	< 0.05
Systolic blood pressure (mmHg)	122.2 (± 10.87)	123.7 (± 13.72)	< 0.05
Medical reimbursement (%)	80	90	< 0.05

After PSM, as shown in Table 2, there were no significant differences in baseline characteristics between exposed (conventional treatment + salvianolate injection) group and unexposed (conventional treatment + salvianolate injection) group.

**Table 2:** Baseline characteristics after propensity score matching

Characteristic	Conventional treatment + salvianolate injection (n = 401)	Conventional treatment (n = 401)	*P*
Age (years)	64.2 (± 12.2)	65.8 (± 12.2)	0.069
Male sex (%)	60	60	1
Diastolic blood pressure (mmHg)	71.3 (± 6.5)	71.1 (± 6.3)	0.724
Systolic blood pressure (mmHg)	122.7 (± 10.9)	122.6 (± 11.8)	0.825
Medical reimbursement (%)	90	90	1
Dosage of nitrates (mg)	19.3 (± 123.5)	19.3 (± 123.5)	1
Administration of nitrates^a^	0.2	0.2	1

^a^Using only oral nitrates was set as 0; using only nitrates injections was set as 1; using both dosage forms was set at 2

### Cost-consequence analysis of overall cohort

For the overall cohort, as shown in Table 3, hospital stay in exposed group was significantly decreased by 2.9 days compared with unexposed group (11.7 [± 7.5] vs 14.6 [± 1.5] days; *P* < 0.05). Total nitrates dosage was significantly decreased by 172.4 mg in exposed group compared with unexposed group (457.7 [± 511.4] vs 630.1 [± 650.4 mg; *P* < 0.05). A decrease of 2636.4 CNY in total medical costs was found in patients who received combination of conventional treatment and salvianolate injection (14,726.3 [± 18,165.8] vs 17,362.7 [± 22,161.8] CNY; *P* = 0.054), although there was no significant difference between exposed group and unexposed group at *P* < 0.05 level.

**Table 3:** Comparison of clinical and economic outcomes in overall cohort

Outcome	Conventional treatment + salvianolate injection (n = 401)	Conventional treatment (n = 401)	Difference	*P*
Hospital stay (days)	11.7 (± 7.5)	14.6 (± 11.5)	− 2.9	< 0.05
Total nitrates dosage (mg)	457.7 (± 511.4)	630.1 (± 650.4)	− 172.4	< 0.05
Total medical costs (CNY)	14,726.3 (± 18,165.8)	17,362.7 (± 22,161.8)	− 2636.4	0.054
Subcategories costs (CNY)				
Supplies cost	5538.6 (± 13,486.6)	5770.9 (12,458.5)	− 232.3	0.801
Pharmacy cost	3122.5 (± 3124.2)	4890.1 (± 5218.0)	− 1767.6	< 0.05
Examination cost	1410.6 (± 1277.5)	1954.9 (± 2427.2)	− 544.3	< 0.05
Laboratory cost	1351.7 (± 818.3)	1564.2 (± 1330.3)	− 212.5	< 0.05
Operation cost	2626.2 (± 1884.2)	4223.2 (± 3211.2)	− 1597.0	< 0.05
Treatment cost	447.4 (± 831.6)	1005.7 (± 1687.1)	− 558.3	< 0.05
Chinese patent drug cost	1316.6 (± 581.0)	141.7 (± 474.6)	1174.9	< 0.05

As for subcategories costs, in exposed group, pharmacy cost, examination cost, laboratory cost, operation cost, and treatment cost were significantly (*P* < 0.05) lower, while Chinese patent drug cost was significantly (1316.6 [± 581.0] vs 141.7 [± 474.6] CNY; *P* < 0.05) higher. Supplies cost did not significantly (*P* = 0.801) differ between exposed group and unexposed group.

**Fig. 2:**
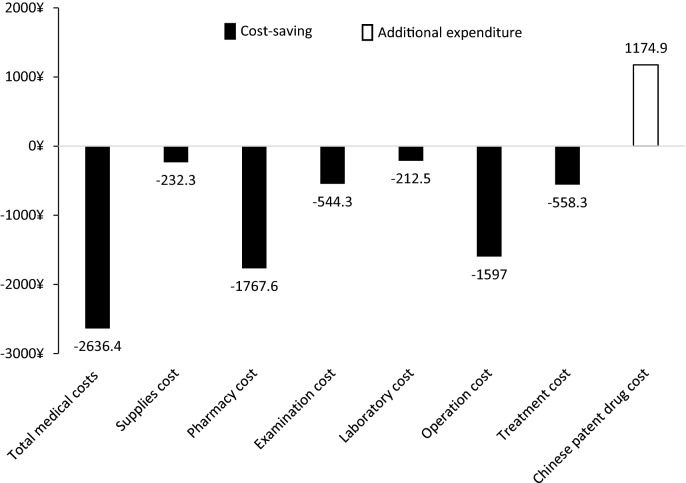
Cost-saving and additional expenditure in overall cohort

As presented in Fig. 2, compared with unexposed group, exposed group had cost-saving in total medical costs and subcategories costs except Chinese patent drug cost. However, even with additional expenditure of 1174 CNY for Chinese patent drug, there was still 2636.4 CNY cost saving in total medical costs for exposed group.

### Cost-consequence analysis of chronic ischemic heart disease subcohort

For the chronic ischemic heart disease subcohort, the results of cost-consequence analysis is reported in Table 4. Hospital stay in exposed group was 11.9 (± 7.7) days, which was significantly 3.6 days less than 15.5 (± 13.0) days of unexposed group (*P* < 0.05). Total nitrates dosage of patients in exposed group was significantly 250.7 mg lower than that of unexposed group (457.7 [± 511.4] vs 703.4 [± 845.2] mg; *P *< 0.05). Comparison of total medical costs indicated a significant cost saving of 4339.5 CNY in exposed group (12,694.9 [± 20,609.5] vs 17,034.4 [± 21,251.3] CNY; *P* < 0.05).

**Table 4:** Comparison of clinical and economic outcomes in subcohort of chronic ischemic heart disease

Outcome	Conventional treatment + salvianolate injection (n = 129)	Conventional treatment (n = 129)	Difference	*P*
Hospital stay (d)	11.9 (± 7.7)	15.5 (± 13.0)	− 3.6	< 0.05
Total nitrates dosage (mg)	452.7 (± 490.1)	703.4 (± 845.2)	− 250.7	< 0.05
Total medical costs (CNY)	12,694.9 (± 20,609.5)	17,034.4 (± 21,251.3)	− 4339.5	< 0.05
Subcategories costs (CNY)				
Supplies cost	3760.4 (± 15,171.77)	5158.4 (± 9740.56)	− 1398	0.382
Pharmacy cost	3308.2 (± 3660.5)	4925.7 (± 5065.2)	− 1617.5	< 0.05
Examination cost	1551.1 (± 1403.78)	1957.2 (± 1742.96)	− 406.1	< 0.05
Laboratory cost	1365.6 (± 823.26)	1627.4 (± 1384.94)	− 261.8	0.07
Operation cost	2592.7 (± 2243.94)	4705.1 (± 3228.53)	− 2112.4	< 0.05
Treatment cost	462 (± 895.68)	1042 (± 1678.62)	− 580	< 0.05
Chinese patent drug cost	1334.9 (± 637.9)	145.6 (± 378.8)	1189.3	< 0.05

Among the subcategories costs, pharmacy cost, examination cost, operation cost, and treatment cost were significantly (*P* < 0.05) lower in exposed group, while patients in exposed group incurred significantly (*P* < 0.05) more Chinese patent drug cost. Supplies cost (*P* = 0.382) and laboratory cost (*P* = 0.007) were not significantly different between exposed group and unexposed group.

**Fig. 3:**
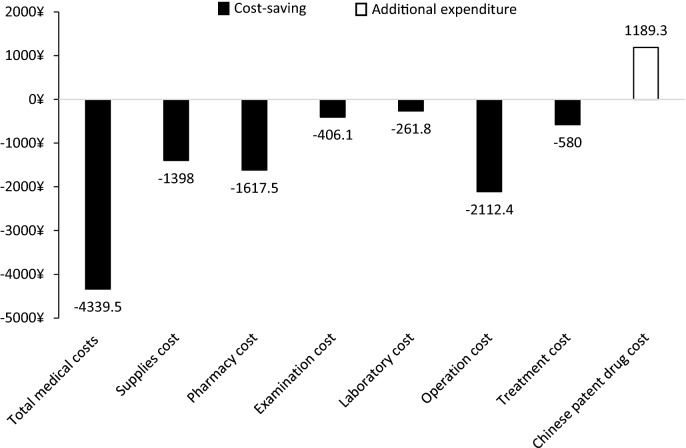
Cost-saving and additional expenditure in chronic ischemic heart disease subcohort

As shown in Fig. 3, there was obvious cost saving for exposed group in supplies cost (1398 CNY), pharmacy cost (1617.5 CNY) and operation cost (2112.4 CNY). Although the combination use of conventional treatment and salvianolate injection caused additional expenditure of Chinese patent drug (1189.3 CNY), it still resulted in 4339.5 CNY saving of total medical costs compared with unexposed group.

### Sensitivity analysis

The results of the sensitivity analysis for PSM showed that, within either the overall cohort or subcohort, the study results were insensitive to whether patients receiving nitrate medications, nitrate base usage, and what caliper values were used for PSM. The results were thus approved to be robust.

For sensitivity analysis for statistics methods, the results showed that the test results of the total medical costs difference were sensitive to the choice of statistical methods: the results obtained by using *t* test were not significant, while the results obtained using Wilcoxon rank-sum test were mostly significant at the 95% level. Since the data of total medical costs did not follow a normal distribution, it tended to accept non-parametric test results.

## Discussion

Economic value is an essential component in the assessment for a drug applied in clinic [22, 23]. Especially for TCM products, economic evaluation is becoming more and more important for their use and imbursement and may even influence their international market access [24]. This study compared the cost-consequence of salvianolate injection combined with conventional treatment and conventional treatment alone. The result provides a reference for doctors who used salvianolate injection to achieve the best balance between the effectiveness and economic value of salvianolate injection for CHD. At the same time, it also provides a reference for policy marker to make decisions about including salvianolate injection into drug formulary to promote rational use of drugs in the clinical practice for CHD.

For the purpose of pharmacoeconomic evaluation, data obtained in real world is considered more instructive than data extracted from clinical trials [25]. In this study, we retrospectively analyzed the CHD treatment in electronic medical record database. This study setting was similar to real world. It is indicated that real world study design could bring about many external confounding factors [26]. To control confounding factors and reduce bias, a PSM analysis was performed in this study to balance baseline characteristics between exposed group and unexposed group. Therefore, the study results provided reliable information of cost-consequence about salvianolate injection for CHD treatment.

Our findings showed a shorter hospital stay and a lower nitrates dosage observed in exposed group relative to unexposed group, for both overall CHD cohort and subcohort of chronic ischemic heart disease. Hospital stay and nitrates dosage were generally regarded as two representative indicators for CHD treatment [27–29]. Therefore, the shorter hospital stay and lower nitrate dosage could be regarded as indirect reflections of a better clinic effectiveness of the combined use of salvianolate injection and conventional treatment.

In addition to clinical consequence of shorter hospital stay and lower nitrates dosage, cost savings of total medical costs and subcategories costs (except Chinese patent drug cost) were observed in exposed group. The increase of Chinese patent drug cost was partially due to use of salvianolate injection. However, such increase in costs was offset by cost savings associated with the improvement of health status with the use of salvianolate injection. For overall cohort, salvianolate injection saved 2636.4 CNY total medical costs per patient admission. The result had an obvious economic value, but it was not statistically significant at *P* < 0.05. Accordingly, a further research with larger sample size will be need to reconfirm the conclusion. For chronic ischemic heart disease subcohort, the cost savings were more notable (4339.5 CNY). It indicated that salvianolate injection had a better cost consequence in this subcohort of chronic ischemic heart disease.

Nevertheless, some limitations of this study deserve to be mentioned for future research. First, we analyzed the patients who were admitted to hospital between 2011 and 2015. The long-time span might give rise to a time-dependent bias. Second, we extracted the data from a single hospital, so the generalizability of the results might be limited. Hence, a further study based on a bigger medical record data from more hospitals is warranted. Third, because of the limited data in the original medical record, this study did not include CHD severity and complications into analysis. While PSM used in this study can increase the comparison reliability to a great extent, future study using more comprehensive data about CHD will provide further information about cost-consequence of salvianolate injection for treatment of subcategory of CHD.

## Conclusions

Compared with conventional treatment for CHD, combination of salvianolate injection and conventional treatment was associated with a reduction in hospital stay and total nitrates dosage. The acquisition cost of Chinese patent drug (including salvianolate injection) was offset by a higher reduction in total medical costs, especially for patients with chronic ischemic heart disease.

## Additional file

**Additional file 1.** Minimum standards of reporting checklist.

## Abbreviations

CHD: coronary heart disease
PSM: propensity score matching
TCM: traditional Chinese medicine
